# Gut Microbiome Signatures Are Predictive of Cognitive Impairment in Hypertension Patients—A Cohort Study

**DOI:** 10.3389/fmicb.2022.841614

**Published:** 2022-04-08

**Authors:** Lei Qu, Zhouyan Dong, Songcui Ma, Yaping Liu, Wei Zhou, Zitong Wang, Chen Wu, Rui Ma, Xinze Jiang, Tingting Zu, Mei Cheng, Yulong Wu

**Affiliations:** ^1^Department of Pathogenic Biology, Binzhou Medical University, Yantai, China; ^2^Yantai Yuhuangding Hospital, Yantai, China; ^3^Institute of Health and Disease Management, Binzhou Medical University, Yantai, China; ^4^ School of Nursing and Rehabilitation, Shandong University, Jinan, China; ^5^Clinical Medicine School, Binzhou Medical University, Yantai, China

**Keywords:** gut microbiota, hypertension, cognitive impair, prediction model, 16S/18S ribosomal RNA gene analysis

## Abstract

Growing evidence has demonstrated that hypertension was associated with dysbiosis of intestinal flora. Since intestinal microbes could critically regulate neurofunction *via* the intestinal–brain axis, the study aimed to reveal the role and prediction value of intestinal flora alteration in hypertension-associated cognitive impairment. A cohort of 97 participants included 63 hypertension patients and 34 healthy controls. The structure of intestinal flora was analyzed by V3–V4 16S rRNA amplicon sequencing. The cognitive function was assessed using the Montreal Cognitive Assessment (MoCA) scale, and 31 patients were considered to have cognitive impairment (MoCA < 26). Patients with cognitive impairment had considerable alterations in intestinal flora structure, composition, and function compared with normal-cognitive patients. In particular, the abundance of LPS-containing taxa (Proteobacteria, Gammaproteobacteria, Enterobacterales, *Enterobacteriaceae*, and *Escherichia–Shigella*) and SCFA-producing taxon (*Prevotella*) significantly changed in cognition-impaired patients. Tax4Fun predication results showed downregulation of glycan biosynthesis and metabolism in hypertension patients with cognitive impairment. Additionally, the pathway was demonstrated to be significantly correlated with LPS-containing taxa (Proteobacteria, Gammaproteobacteria, Enterobacterales, *Enterobacteriaceae*, and *Escherichia–Shigella*) and SCFA-producing taxon *Prevotella*. Furthermore, the taxa-based multiple joint prediction model (9×) was demonstrated to have excellent diagnostic potential for cognitive impairment of hypertension patients (AUC = 0.944). The current study revealed the involvement of intestinal microbiota dysbiosis in cognition-impaired hypertension patients and provided an objective predictive index for this cognition disorder.

## Introduction

Hypertension is a worldwide public health issue. A recent study reported that the number of adults aged 30–79 with hypertension has increased from 650 million to 1.28 billion in the past 30 years (NCD Risk Factor Collaboration, 2021). It is well known that hypertension could lead to multi-system complications, including heart failure, chronic kidney disease, and mild cognitive impairment. Noteworthy, epidemiological studies found that hypertension is an important risk factor of cognitive function impairment, which significantly increased the incidence of Alzheimer’s disease (AD) and vascular dementia (Qiu et al., 2005; Abete et al., 2014). Previous studies revealed that the neuropathological features of AD, such as the number of amyloid plaques and nerve fiber tangles, were significantly increased in the cerebral cortex and hippocampus of patients with chronic hypertension (Petrovitch et al., 2000). To date, effective or disease-modifying drugs against AD are not available since the pathological factors of AD are still unclear. The lack of efficient therapy for AD has put forward the emphasis of treatments on people at risk of dementia, such as mild cognitive impairment condition (Akbari et al., 2016). Hence, it is necessary to investigate the pathogenesis of mild cognitive impairment induced by hypertension, thereby promoting strategies for this cognitive decline.

Recently, ample research has solidly proved the association of intestinal flora dysbiosis with hypertension. Yang et al. (2015) reported that the abundance and diversity of intestinal flora in hypertension patients were significantly decreased, and the structure of intestinal flora was significantly different from those of healthy people. Besides, specific metabolic pathways were considered to be relevant to hypertension (Koh et al., 2016; Sawicki et al., 2017). It is worth noting that specific functions of intestinal flora, such as those involved in short-chain fatty acid (SCFA) metabolism (Huart et al., 2019; Oyama and Node, 2019; Yang F. et al., 2020; Wu et al., 2021) and lipopolysaccharide (LPS) content (Lorenzoni and Wideman, 2008; Mell et al., 2015; Yan et al., 2017; Dubinski et al., 2021), were found to be associated with both hypertension and certain cognitive impairment diseases. Cumulative evidence indicated the pivotal role of intestinal flora in regulating brain function through the microbe–gut–brain axis (Westfall et al., 2017; Zhu et al., 2017). Imbalance of intestinal flora can lead to nervous diseases such as AD (Liu et al., 2019), ischemic stroke (Ling et al., 2020; Liu et al., 2020), and Parkinson’s disease (Sampson et al., 2016; Pietrucci et al., 2019). Hence, we hypothesized that dysbiosis of the gut microbiota might be involved in the process of hypertension-associated cognitive impairment.

The present study investigated the alteration of the intestinal microbiota and cognition by 16S rRNA amplicon sequencing and Montreal Cognitive Assessment, respectively. Moreover, we analyzed the correlation of the significantly changed specific intestinal taxa with the score of cognitive function scale among hypertension patients. Furthermore, we established a bacteria-based biological predictive model for hypertension-associated cognitive impairment, which enables sensitive diagnosis of cognitive impairment of hypertension patients, and therefore facilitates the potential novel therapy targeting at regaining gut microbiota homeostasis.

## Materials and Methods

### Study Cohort and Patient Characteristics

Hypertension patients were recruited from the Affiliated Hospital of Binzhou Medical University, Yantai, China, during December 2019 to June 2020. The research was approved and supervised by the Institution Review Board of Binzhou Medical University (No. 2018-085). Each subject signed a written informed consent voluntarily before enrollment. Every participant enrolled in the hypertension patients group met the following criteria: According to Chinese Guidelines for Prevention and Treatment of Hypertension (2018 Revised Version) (Joint Committee for Guideline Revision, 2019), (i) Without using antihypertensive drugs, systolic blood pressure ≤ 140 mmHg and/or diastolic blood pressure ≤ 90 mmHg; (ii) With a history of hypertension, and using antihypertensive drugs at present, even if blood pressure < 140/90 mmHg were included. Meanwhile, spouses of hypertension patients were voluntarily included as the Control group, which aimed to mitigate the impact of dietary and lifestyle habits in blood pressure. The exclusion criteria included the following: (i) current or history of neurological or chronic psychiatric disorder, schizophrenia, brain injury or stroke, major depressive disorder, or severe anxiety disorders requiring pharmacotherapy; (ii) suffering from digestive system diseases, diabetes, respiratory diseases, nervous system diseases such as AD and Parkinson’s disease, or other severe primary diseases; (iii) with severe hearing, and visual or motor deficits that may interfere with cognitive tests; (iv) treatment with antibiotics, probiotics, or prebiotics within 1 month; and (v) current or history of substance dependence. Ultimately, 63 hypertension patients and 34 healthy people were recruited in our research.

### Cognitive Function Assessment

The Chinese versions of the Montreal Cognitive Assessment (MoCA) translated by Yu et al. (2012) was used to assess cognitive function (Nasreddine et al., 2005), which consists of seven domains of cognitive functional performance including visual space and executive function, naming, attention, language, abstract thinking, delayed memory, and orientation. Cronbach’s α coefficient of this MoCA scale is 0.88. The total score is 30 points (adding one point to the MoCA total score of individuals with ≤ 12 years of education). A score of MoCA ≥ 26 has been identified as “normal” cognition, and a score of MoCA < 26 indicates cognitive impairment (Nasreddine et al., 2005; Copersino et al., 2009).

### V3–V4 16S rRNA Gene Sequencing Analysis

Fecal samples were freshly collected and stored at –80°C for subsequent sequencing analysis. Total genome DNA from samples was extracted using CTAB protocol. 16S rRNA genes’ V3–V4 regions were amplified used specific forward primer 341F (5′-CCTAYGGGRBGCASCAG-3′) and reverse primer 806R (5′-GGACTACNNGGGTATCTAAT-3′) with the barcode. All PCR reactions were carried out with Phusion ^®^ High-Fidelity PCR Master Mix (New England Biolabs, Hitchin, United Kingdom). Thermal cycling consisted of initial denaturation at 98°C for 1 min, followed by 30 cycles of denaturation at 98°C for 10 s, annealing at 50°C for 30 s, and elongation at 72°C for 30 s, and, finally, 72°C for 5 min. Then, a mixture of PCR products was purified with Qiagen Gel Extraction Kit (Qiagen, Hilden, Germany). TruSeq ^®^ DNA PCR-Free Sample Preparation Kit (Illumina, San Diego, CA, United States) was used to generate sequencing libraries. The library quality was assessed on the Qubit@ 2.0 Fluorometer (Thermo Fisher Scientific, MA, United States) and Agilent Bioanalyzer 2100 system (Agilent, Santa Clara, CA, United States), then sequenced on an Illumina NovaSeq6000 platform (Novogene, Cambridge, United Kingdom) and 250-bp paired-end reads were generated. Sequence analyses were performed by Uparse software (version 7.0.1001) (Edgar, 2013), which, with ≥ 97% similarity, were assigned to the same OTUs. For each representative sequence, the SSUrRNA of the SILVA database (version 138.1) (Quast et al., 2013) was used based on Mothur software (version 1.46.1) to annotate taxonomic information (Schloss et al., 2009). In order to study phylogenetic relationship of different OTUs, and the difference of the dominant species in different samples or groups, multiple sequence alignment was conducted using the MUSCLE software (version 3.8.31) (Edgar, 2004). QIIME software (version 1.9.1) was employed to calculate the alpha and beta diversity. Venn diagrams and rarefaction curves were plotted using R software (version 2.15.3). Principal coordinate analysis (PCoA) was performed to obtain principal coordinates and visualize complex multidimensional data, which were displayed by WGCNA package, stat packages, vegan package, and ggplot2 package in R software (version 2.15.3) (Zuo et al., 2021). Anosim analysis was performed using vegan package in R software (version 2.15.3).

### Analysis of Composition and Function Differences

In order to find species with significant differences among groups, MetaStat analysis was performed using R software (version 2.15.3), and the relative abundance differences of intestinal flora at the level of phylum, class, order, family, and genus were compared. LEfSe software (version 1.0) was used for linear discriminant analysis effect size analysis, and the linear discriminant analysis (LDA) value was set to 4 (Segata et al., 2011). Based on the 16S SILVA database, the function of intestinal flora prediction was analyzed by Tax4Fun package of R software (Aßhauer et al., 2015). The 16S rRNA gene sequences extracted from KEGG prokaryote genome database^1^ were aligned to the SILVA SSU Ref NR 99 database^2^ by BLASTN algorithm, and then the functional information of KEGG prokaryote genome database was mapped to the SILVA database for functional annotation.

### Statistical Analysis

The data were presented as the mean ± standard deviations (SDs). The distribution data were evaluated by Shapiro–Wilk test. Normally distributed continuous variables were analyzed using one-way analysis of variance (ANOVA) followed by Fisher’s Least Significant Difference (LSD) multiple comparisons. Non-normally distributed continuous variables were assessed by Welch’s ANOVA, and the *post hoc* multiple comparisons were performed using the Games–Howell test. Correlations between intestinal taxa and MoCA performance were analyzed using Spearman or Pearson correlation analysis, as appropriate. To choose the optimal model for diagnosis of cognitive impairment of hypertension patients, receiver operating characteristic (ROC) area under the curve was estimated. The Youden index was used to determine the best cutoff of the ROC curve (Fluss et al., 2005). The statistical significance was determined at *p*-value < 0.05. SPSS 22.0 software (IBM, Chicago, IL, United States) was used to perform the statistical analysis. Images were generated by Adobe Illustrate (version 2018CC) and R (version 4.1.1).

## Results

### Demographic Characteristics and Cognitive Function Analysis

The present study recruited a total of 97 subjects, including 63 hypertension patients and 32 healthy individuals (the Control group). Of the hypertension patients, 31 subjects were determined to have cognitive impairment (MoCA < 26) (the MCI group), while 32 hypertension patients were determined to have no cognitive impairment (the NMCI group). The demographic characteristics of the MCI group, NMCI group, and Control groups are presented in Table 1. There were no significant differences in age, gender, education years, and BMI indexes (*p* > 0.05).

**TABLE 1:** Comparison of demographic and clinical characteristics of subjects.

Variables	Hypertension patients (*n* = 63)		Control (*n* = 34)
	Cognitive impairment (*n* = 31)	No cognitive impairment (*n* = 32)	
Age (years, mean ± SD)	60.52 ± 4.84	59.13 ± 4.35	59.15 ± 6.21
Female ratio (%)	17 (54.80)	14 (43.80)	16 (47.10)
Education (years, mean ± SD)	8.03 ± 2.73	8.66 ± 2.98	7.41 ± 1.69
BMI (kg/m^2^, mean ± SD)	25.70 ± 2.90	26.11 ± 2.99	24.82 ± 2.28
SBP (mmHg)	157.03 ± 19.50**	161.03 ± 21.25**	123.67 ± 5.83
DBP (mmHg)	89.41 ± 12.72*	93.48 ± 12.93**	77.31 ± 7.90

BMI, body mass index; SD, standard deviation; SBD, systolic blood pressure; DBP, diastolic blood pressure. *p < 0.05, **p < 0.01, compared with the Control group.

Montreal Cognitive Assessment results are shown in Table 2; the total score of MoCA and the scores of specific function domains including visuospatial and executive function, attention, language, abstraction, delayed recall, and orientation were significantly lower in the MCI group than the NMCI group (*p* < 0.05), while there was no significant difference in naming domain among three groups (*p* > 0.05).

**TABLE 2:** Comparison of MoCA score.

Variables	Hypertension patients (*n* = 63)		Control (*n* = 34)
	Cognitive impairment (*n* = 31)	No cognitive impairment (*n* = 32)	
MoCA total score	22.52 ± 1.88**^##^	26.97 ± 0.82	27.09 ± 0.83
Visuospatial and executive function	3.32 ± 0.70**^##^	4.25 ± 0.62	4.21 ± 0.73
Naming	2.97 ± 0.18	3.00 ± 0.00	3.00 ± 0.00
Attention	3.94 ± 0.81**^##^	5.44 ± 0.91	5.41 ± 0.92
Language	2.29 ± 0.78*^##^	2.72 ± 0.52	2.82 ± 0.39
Abstraction	1.74 ± 0.44**^##^	1.97 ± 0.18	2.00 ± 0.00
Delayed recall	2.87 ± 0.62**^##^	3.66 ± 0.65	3.65 ± 0.73
Orientation	5.39 ± 0.88**^##^	5.94 ± 0.25	6.00 ± 0.00

*All results were displayed as: mean ± standard deviation.*

*p < 0.05, **p < 0.01, compared with the NMCI group.

*^##^p < 0.01, compared with the Control group.*

### Diversity and Functional Difference of Intestinal Flora

#### Operational Taxonomic Units Cluster Analysis

**FIGURE 1:**
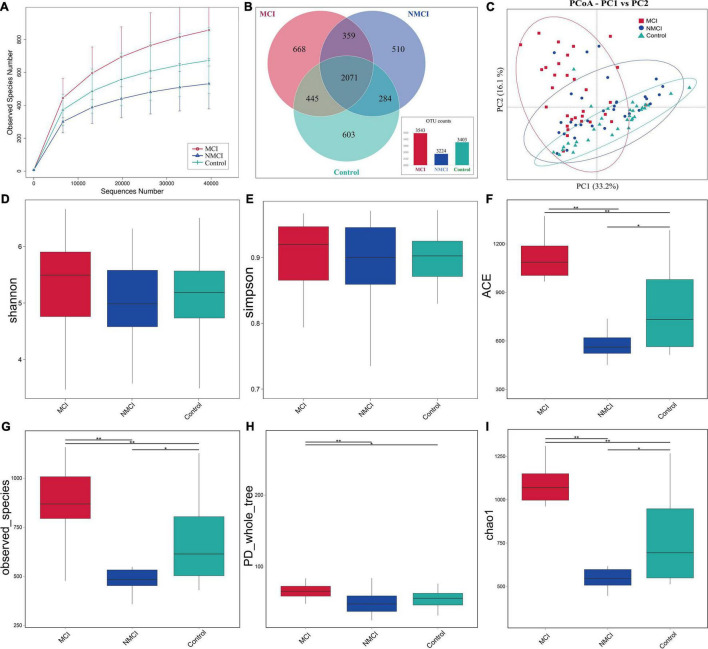
Comparison of the intestinal microbiota richness and diversity among three groups. **(A)** Rarefaction curve showed the number of identified OTUs approached a plateau, indicated that the sequencing depth is enough for analysis. **(B)** Venn diagram showing the shared and unique OTUs. **(C)** PCoA based on weighted UniFrac distance was used to analyze the structure of intestinal flora among three groups (Anosim, *p* < 0.01): each dot represents a sample, and different colors and shapes indicate the group to which they belong. The result is shown along the first and second axes of the PCoA plot. Numbers in parentheses represent explained variation. Ellipses represent different groups. Shannon **(D)**, Simpson **(E)**, ACE **(F)**, observed_Species **(G)**, PD_whole_tree **(H)**, and Chao1 **(I)** indexes were used to assess alpha diversity. MCI, hypertension patients with cognitive impairment, NMCI, hypertension patients without cognitive impairment; Control, health control subjects.

The raw data were analyzed, followed by splicing, quality control, and chimeric filtration. Ultimately, 9,278,977 reads were generated, and 96.84% (8,985,741) passed the quality control screening. These reads were clustered to 4,940 operational taxonomic units (OTUs). The rarefaction curve for the three groups shows that the number of identified OTUs approached a plateau (Figure 1A), which means that the sequencing depth is enough for analysis. Venn diagram showed that the number of unique OTUs in the MCI group, NMCI group, and Control group was 668, 510, and 603, respectively (Figure 1B).

#### Alpha Diversity Analysis

Alpha diversity was evaluated by Chao1, ACE, observed_Species, PD_whole_tree, Shannon, and Simpson indexes. As shown in Figures 1D–I and Table 3, compared with the Control group, Chao1, ACE, and observed_species were highly significantly lower in the NMCI group (*p* < 0.01). It indicated that microbial community abundance and diversity were significantly lower in hypertension patients than healthy individuals.

**TABLE 3:** Comparison of alpha diversity.

Variables	Hypertension patients (*n* = 63)		Control
	Cognitive impairment (*n* = 31)	No cognitive impairment (*n* = 32)	
Shannon	5.320	5.002	5.136
Simpson	0.895	0.896	0.894
Chao1	1,022.603**^##^	614.790^##^	772.703
ACE	1,047.694**^##^	632.768^##^	793.967
observed_species	857**^##^	531^##^	672
PD_whole_tree	70.414**^##^	49.865	54.902

All results were displayed as: mean ± standard deviation. **p < 0.01, compared with the NMCI group; ^##^p < 0.01, compared with the Control group.

Compared with the NMCI group, Chao1, ACE, observed_species, and PD_whole_tree indexes were significantly higher in the MCI group (*p* < 0.01), indicating a significantly higher microbial community abundance and diversity in cognition-impaired patients than normal-cognition patients. The Shannon and Simpson indexes were lower in the MCI group than the NMCI group, although the difference was not significant.

#### Beta Diversity Analysis

Principal coordinate analysis based on weighted UniFrac distance was used to analyze the structure of intestinal flora among three groups (Anosim, *p* < 0.01) (Figure 1C). There was a significant separation trend in the direction of the first principal component axis (PC1) and the second principal component axis (PC2). The first principal component axis (PC1) contribution was 33.2%, and that of the second principal component axis (PC2) was 16.1%.

### Differences in the Composition and Function of Intestinal Flora

**FIGURE 2:**
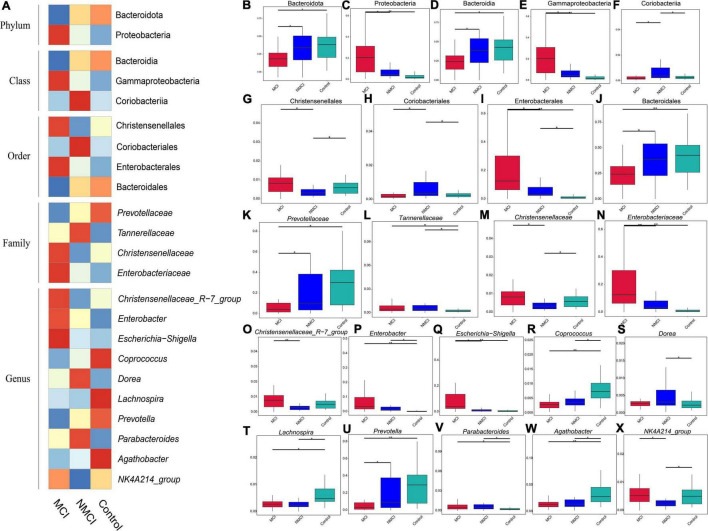
Differences in the abundance of intestinal flora. MetaStat analysis was performed to compare the relative abundance differences of intestinal flora. **(A)** Heatmap of relative abundance difference. **(B,C)** Phylum level. **(D–F)** Class level. **(G–J)** Order level. **(K–N)** Family level. **(O–X)** Genus level. MCI, hypertension patients with cognitive impairment; NMCI, hypertension patients without cognitive impairment; Control, health control subjects.

MetaStat analysis and LDA effect size (LEfSe) analyses were performed to assess the difference in abundance and composition among the three groups. Figure 2A showed a summarized relative abundance of microbial taxa, Figures 2B–X displayed detailed relative abundance of each taxon.

**FIGURE 3:**
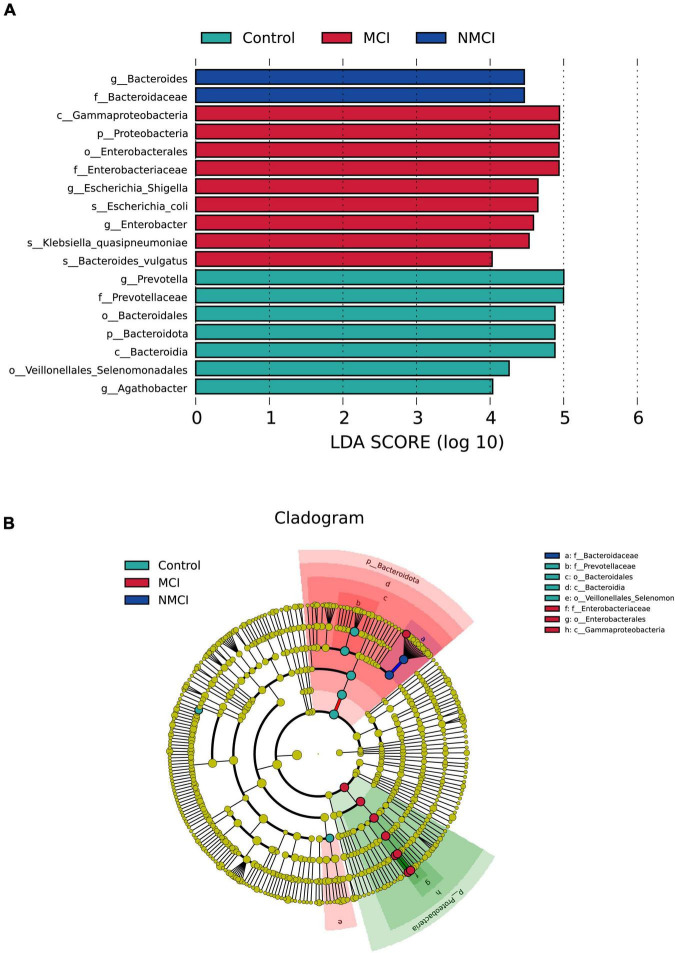
Differences in the composition of intestinal flora. Linear discriminant analysis (LDA) effect size (LEfSe) analyses were performed to assess the difference in composition among the three groups. **(A)** LDA results showed the distinct taxa. **(B)** LEfSe results indicating the phylogenetic distribution of microbiota. Lowercase letters represent different classification levels; p, phylum; c, class; o, order; f, family; g, genus. MCI, hypertension patients with cognitive impairment; NMCI, hypertension patients without cognitive impairment; Control, health control subjects.

The results of MetaStat analysis showed that the relative abundance of 9 taxa were significantly higher in the MCI group than in the NMCI group, namely, Proteobacteria, Gammaproteobacteria, Enterobacterales, Christensenellales, *Christensenellaceae*, *Enterobacteriaceae*, *Christensenellaceae_R-7_Group*, *Escherichia-Shigella*, and *NK4A214_group* (21.159% vs. 9.006%, 21.104% vs. 8.843%, 19.753% vs. 7.512%, 0.955% vs. 0.438%, 0.955% vs. 0.438%, 19.736% vs. 7.491%, 0.911% vs. 0.364%, 11.40% vs. 1.97%, 0.579% vs. 0.320%; *p* < 0.01) (see Figures 2C,E,G,I,M–O,Q,X). Besides, the relative abundance of 7 groups was significantly lower in the MCI group than the NMCI group, which were Bacteroidota, Bacteroidia, Coriobacteriia, Coriobacteriales, Bacteroidales, *Prevotellaceae*, and *Prevotella* (24.800% vs. 37.094%, 24.800% vs. 37.094%, 0.282% vs. 0.757%, 0.282% vs. 0.757%, 24.784% vs. 37.053%, 8.168% vs. 19.128%, and 7.202% vs. 18.541%; *p* < 0.01) (see Figures 2B,D,F,H,J,K,U). LEfSe analysis was used to identify the key phenotypes, according to LDA scores, Phylum: Proteobacteria; Class: Gammaproteobacteria; Order: Enterobacterales; Family: *Enterobacteriaceae*; Genus: *Escherichia–Shigella*, *Enterobacter*; Species: *Klebsiella quasipneumoniae*, *Bacteroides vulgatus*, and *Escherichia coli* were distinct in the MCI group (see Figures 3A,B).

**FIGURE 4:**
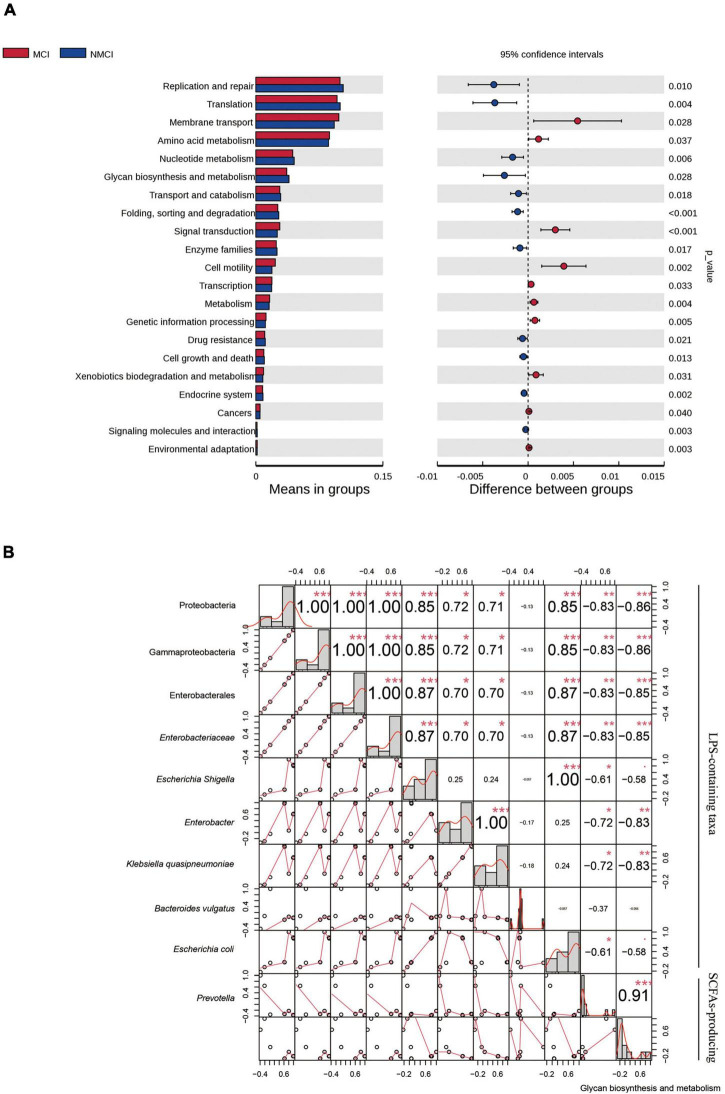
Function prediction and correlation analysis. **(A)** Function predication was inferred by using Tax4Fun package of R software, and 24 KEGG-level-2 pathways were altered. **(B)** Pearson correlations among the glycan biosynthesis and metabolism pathway and distinct taxa. Numbers indicated correlation coefficients, and size of points reflected the strength of the correlation. *p < 0.05, **p < 0.01, ***p < 0.001. MCI, hypertension patients with cognitive impairment; NMCI, hypertension patients without cognitive impairment; Control, health control subjects.

To predict the functions of intestinal microflora, Tax4Fun analysis was performed, as shown in Figure 4A, at the second level of KEGG; 24 pathways, such as glycan biosynthesis and metabolism, transcription, environmental adaptation, replication and repair, and amino acid metabolism, were enriched differently between the MCI group and the NMCI group. Specifically, compared with the NMCI group, the glycan biosynthesis and metabolism were significantly weakened (*p* < 0.05). This pathway was significantly negatively associated with the relative abundance of Proteobacteria (*p* < 0.001), Gammaproteobacteria (*p* < 0.001), Enterobacterales (*p* < 0.001), *Enterobacteriaceae* (*p* < 0.001), *Escherichia-Shigella* (*p* < 0.05), *Enterobacter* (*p* < 0.01), and *Klebsiella quasipneumoniae* (*p* < 0.01) and significantly positively associated with the relative abundance of *Prevotella* (*p* < 0.001, Figure 4B).

### Correlation Analysis of Intestinal Taxa With Differential Abundance and Cognitive Function Domains

The result showed that there were nine intestinal microbiota (Proteobacteria, Gammaproteobacteria, Enterobacterales, *Enterobacteriaceae*, Christensenellales, *Christensenellaceae*, *Christensenellaceae_R-7_group*, *Escherichia-Shigella*, and *NK4A214_group*) that were significantly negatively correlated to the MoCA total score (*p* < 0.01), and two intestinal flora microbiota (Coriobacteriia and Coriobacteriales) that were highly positively correlated with the total score of the MoCA (*p* < 0.01). Our results indicated that these nine specific taxa (e.g., Proteobacteria, Gammaproteobacteria, and Enterobacterales) showed a potential possibility of cognitive impairment in hypertension patients, whereas Coriobacteriia and Coriobacteriales might be beneficial to the cognition of hypertension patients.

**FIGURE 5:**
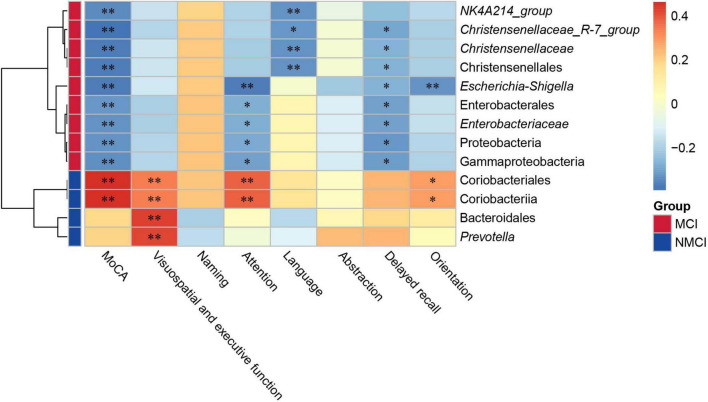
Correlation analysis between gut microbial and MoCA score. The depth of the color in the heatmap represented the strength of the correlation: red represents a positive correlation, whereas blue indicates a negative correlation. Group scale represented enrichment of selected taxa, blue color indicates taxa enriched in the NMCI group, orange color represents taxa enriched in the MCI group. *p < 0.05, ^**^p < 0.01. MCI, hypertension patients with cognitive impairment; NMCI, hypertension patients without cognitive impairment; Control, health control subjects.

In particular, according to our results shown in Figure 5, the attention domain was significantly associated with Proteobacteria (*p* < 0.05), Gammaproteobacteria (*p* < 0.05), Enterobacterales (*p* < 0.05), *Enterobacteriaceae* (*p* < 0.05), Coriobacteriia (*p* < 0.01), Coriobacteriales (*p* < 0.01), and *Escherichia–Shigella* (*p* < 0.01); orientation domain was significantly associated with Coriobacteriia (*p* < 0.05), Coriobacteriales (*p* < 0.05), and *Escherichia–Shigella* (*p* < 0.01); delayed recall domain was significantly associated with Proteobacteria (*p* < 0.05), Gammaproteobacteria (*p* < 0.05), Christensenellales (*p* < 0.05), *Christensenellaceae* (*p* < 0.05), Enterobacterales (*p* < 0.05), *Enterobacteriaceae* (*p* < 0.05), *Escherichia–Shigella* (*p* < 0.05), and *Christensenellaceae_R-7_group* (*p* < 0.05); visuospatial and executive function domain was highly significantly associated with Coriobacteriia (*p* < 0.01), Coriobacteriales (*p* < 0.01), Bacteroidales (*p* < 0.01), and *Prevotella* (*p* < 0.01); language domain was significantly associated with Christensenellales (*p* < 0.01), *Christensenellaceae_*R-7_group (*p* < 0.05), *Christensenellaceae* (*p* < 0.01), and *NK4A214_group* (*p* < 0.01).

### Discriminant Models of Mild Cognitive Impairment in Hypertension Patients Based on Selected Intestinal Taxa

**FIGURE 6:**
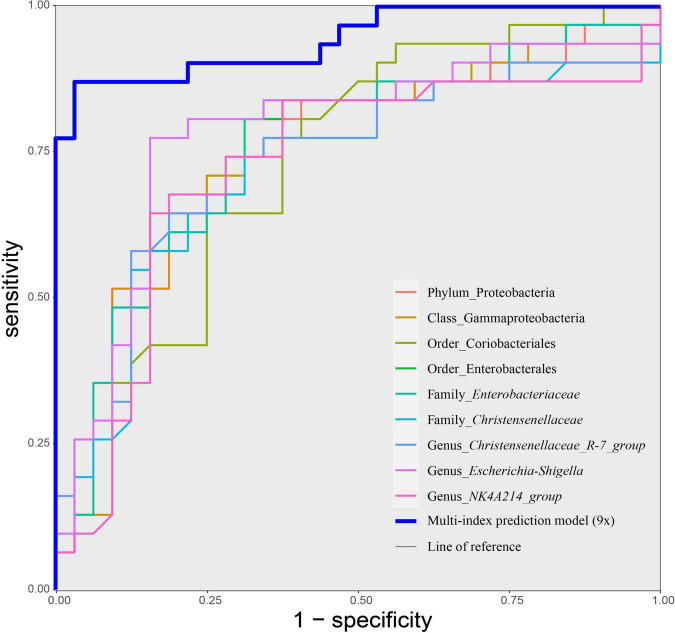
Receiver operating characteristic (ROC) curves of taxa-based single index and the multiple joint model (9×) for discriminating cognitive impairment of hypertension patients. Discriminatory capacity was analyzed by calculating the area under the ROC curve using logistic regression. MCI, hypertension patients with cognitive impairment; NMCI, hypertension patients without cognitive impairment; Control, health control subjects.

Based on the above results, we used 11 candidate microbial groups (Proteobacteria, Coriobacteriia, Gammaproteobacteria, Coriobacteriales, Enterobacterales, Christensenellales, *Enterobacteriaceae*, *Christensenellaceae*, *Christensenellaceae_R-7_group*, *Escherichia-Shigella*, and *NK4A214_group*) significantly related to the total score of MoCA, to build a single index prediction model, separately. ROC analysis was used to assess the diagnostic ability of these indexes for the cognitive impairment of hypertension patients. As shown in Table 4 and Figure 6, the single index based on *Escherichia–Shigella* had the best prediction accuracy, with AUC = 0.783, *p* < 0.01 and Cutoff = 0.025 (Table 4 and Figure 6). To find a more effective prediction model, we built a multi-index joint prediction model based on nine intestinal bacterial taxa (Proteobacteria, Gammaproteobacteria, Coriobacteriales, Enterobacterales, *Enterobacteriaceae*, *Christensenellaceae*, *Christensenellaceae_R-7_group*, *Escherichia-Shigella*, and *NK4A214_group*), which demonstrated better diagnostic values (AUC = 0.944 and Cutoff = 0.584) (Table 4 and Figure 6).

**TABLE 4:** Index prediction models and efficacy evaluation for the risk of mild cognitive impairment in hypertension patients.

Name	AUC (95% CI)	Cutoff value	Sensitivity (%)	Specificity (%)	Accuracy (%)	*p*-value
Proteobacteria	0.748 (0.621–0.875)	0.062	71.4% (25/35)	78.6% (22/28)	74.6% (47/63)	0.001
Coriobacteriia	0.730 (0.604–0.856)	0.003	65.8% (25/38)	76.0% (19/25)	69.8% (44/63)	0.002
Gammaproteobacteria	0.753 (0.628–0.878)	0.061	71.4% (25/35)	78.6% (22/28)	74.6% (47/63)	0.001
Coriobacteriales	0.730 (0.604–0.856)	0.003	65.8% (25/38)	76.0% (19/25)	69.8% (44/63)	0.002
Enterobacterales	0.756 (0.633–0.879)	0.057	71.4% (25/35)	78.6% (22/28)	74.6% (47/63)	<0.01
Christensenellales	0.721 (0.588–0.853)	0.004	68.6% (24/35)	75.0% (21/28)	71.4% (45/63)	0.003
*Enterobacteriaceae*	0.757 (0.634–0.880)	0.051	70.3% (26/37)	80.8% (21/26)	74.6% (47/63)	<0.01
*Christensenellaceae*	0.721 (0.588–0.853)	0.004	68.6% (24/35)	75.0% (21/28)	71.4% (45/63)	0.003
*Christensenellaceae*_*R-7_group*	0.736 (0.607–0.865)	0.004	71.9% (23/32)	74.2% (23/31)	73.0% (46/63)	0.001
*Escherichia*–*Shigella*	0.783 (0.662–0.905)	0.025	82.8% (24/29)	79.4% (27/34)	81.0% (51/63)	<0.01
*NK4A214*_*group*	0.722 (0.586–0.857)	0.004	71.4% (25/35)	78.6% (22/28)	74.6% (47/63)	0.002
Multi-index joint prediction models (9×)	0.944 (0.887–1.000)	0.584	96.4% (27/28)	88.6% (31/35)	76.2% (48/63)	<0.01

*AUC, area under the curve of subject working characteristic; 95% CI, 95% confidence interval; Positive predictive value stands for positive predictive rate; Negative predictive value stands for negative predictive rate. 9×, combination of Proteobacteria, Gammaproteobacteria, Coriobacteriales, Enterobacterales, Enterobacteriaceae, Christensenellaceae, Christensenellaceae_R-7_group, Escherichia-Shigella, and NK4A214_group.*

## Discussion

**FIGURE 7:**
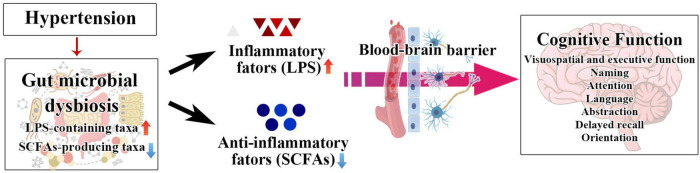
The schematic diagram of our hypothesis about the relationship among SCFA, LPS, hypertension, and cognitive impairment.

Mild cognitive impairment in hypertension patients is at risk of deterioration to dementia. Sensitive diagnosis and thereby early intervention for cognitive impairment caused by hypertension can prevent the incidence of dementia. The present study found that dysbiosis of the intestinal flora is involved in the cognitive dysfunction of hypertension patients. Specifically, LPS-containing taxa (Proteobacteria, Gammaproteobacteria, Enterobacterales, *Enterobacteriaceae*, and *Escherichia–Shigella*) and SCFA-producing taxon (*Prevotella*) were closely correlated with cognitive function of hypertension patients (Figure 7). Moreover, we established multiple joint bacteria-based models for objective diagnosis of cognitive impairment in hypertension patients. The current research is a meaningful effort to characterize the intestinal microbiota of hypertension patients with cognitive impairment and shed new light on the objective diagnosis of cognitive decline in hypertension patients.

Substantial evidence obtained in animals and humans showed that hypertension could induce intestinal flora disorder (Mell et al., 2015; Yang et al., 2015; Marques et al., 2017; Yan et al., 2017). Previous studies indicated that dysbiosis of the intestinal flora is involved in cognitive dysfunction caused by diseases such as Parkinson’s disease and type 2 diabetes (Ren et al., 2020; Zhang et al., 2020). Hence, we hypothesized that the hypertensive-induced dysbiosis of the gut microbiota might play a role in hypertension’s cognitive function decline, which could deteriorate to AD. In the present study, 48.44% of hypertension patients were determined to have cognitive impairment (MoCA < 26). According to Chao1, ACE, observed_species, and PD_whole_tree indexes, we found that the microbial diversity and community abundance significantly rose in cognitive-impaired patients compared to patients with normal cognitive function. The unique OTUs were more frequent in cognitive impairment patients, reaching from 510 in the NMCI group to 668 in the MCI group. Specifically, our results showed that the relative abundance of LPS-containing taxa, Proteobacteria, Gammaproteobacteria, Enterobacterales, *Enterobacteriaceae*, and *Escherichia–Shigella*, was dramatically higher in hypertension patients with cognitive impairment. LPS, as a cell wall component of Gram-negative bacteria, has been previously reported to induce the production of pro-inflammatory cytokines (Oyama et al., 2004). Existing literature has shown that hypertension causes increased permeability of the blood–brain barrier (Faraco et al., 2016). Hence, the gut microbiota-derived products, such as LPS, could leak to the CNS and regulate neuroinflammation, which is a critical mechanism of cognitive impairment (Erny et al., 2015; Rothhammer et al., 2016). A recent clinical study (Liu et al., 2019) demonstrated that Gammaproteobacteria, Enterobacteriales, and *Enterobacteriaceae* showed a progressively enriched prevalence from health control to amnestic mild cognitive impairment and AD patients, which is similar to our results of high relative abundance of LPS-containing taxa in hypertension patients with cognitive impairment. Accumulating studies revealed that cardiovascular diseases such as hypertension and AD have overlapping neuropathological processes (Zlokovic, 2011; Rodrigue et al., 2013; Iadecola, 2016). Hence, we speculate that these specific LPS-containing taxa, including Gammaproteobacteria, Enterobacterales, and *Enterobacteriaceae*, which simultaneously exhibited relatively high abundance in hypertension patients with cognitive impairment and AD patients, may contribute to cognitive deterioration and play a significant role in the neuropathogenesis of hypertension’s cognitive function decline progressive to AD. Further study is required to elucidate the precise role and clarify the mechanism for this phenomenon.

Moreover, we found that the relative abundance of *Prevotella*, which was demonstrated as a SCFA-producing probiotic in the gut (Ren et al., 2021), was significantly decreased in the patients with cognitive impairment compared to the patients without cognitive impairment. Previous evidence demonstrated that downregulation of SCFAs is involved in cognitive impairment diseases including AD, Parkinson’s Disease, congestive heart failure, and sulfamonomethoxine exposure (Ho et al., 2018; Zhu et al., 2019; Sarah et al., 2020; He et al., 2021). SCFAs, such as propionic acid, butyric acid, isobutyric acid, and valeric acid, can inhibit inflammation by regulating histone deacetylase and binding G-protein-coupled receptors (Tan et al., 2014; Yang W. et al., 2020). Our results of Tax4Fun predication showed that the glycan biosynthesis and metabolism were significantly decreased in hypertension patients with cognitive impairment. The trend was supposed to cause carbohydrate metabolism disorder (Woting and Blaut, 2016), resulting in insufficient SCFAs by intestinal flora after digestion of carbohydrate, inducing inflammatory and subsequently affecting cognitive function (Koh et al., 2016; Sawicki et al., 2017). In the present study, we found that the enrichment of glycan biosynthesis and metabolism was significantly positively associated with the SCFA-producing probiotic *Prevotella*, while it was substantially negatively correlated with the relative abundance of LPS-containing taxa (Proteobacteria, Gammaproteobacteria, *Enterobacterales*, *Escherichia–Shigella*, and *Enterobacteriaceae*).

Additionally, we found that the total score of MoCA was strongly associated with the taxa (e.g., Proteobacteria, Coriobacteriales, and *Enterobacteriaceae*), which were significantly altered in the hypertension patients with cognitive impairment. In particular, visuospatial and executive function domain, attention domain, orientation domain, delayed recall domain, and language domain of the MoCA dramatically correlated with selected taxa, respectively. Notably, attention domain, orientation domain, and delayed recall domain were simultaneously correlated with *Escherichia–Shigella*, while attention domain, orientation domain, and visuospatial and executive function domain were consistently associated with Coriobacteriia and Coriobacteriales. Cattaneo et al. (2017) reported that cognitively impaired patients with brain amyloidosis showed a higher abundance of inflammatory bacteria taxon *Escherichia–Shigella* compared with both healthy controls and patients with no brain amyloidosis. The enrichment of *Escherichia–Shigella* is also found to be implicated in post-operative cognitive dysfunction (Lian et al., 2021). Studies investigating the effects of specific bacteria on a distinct domain of cognitive function were still limited. It was revealed by a prospective, longitudinal study that children with a history of bacterial meningitis were at greater risk of impairment in executive ability (Anderson et al., 2004). A study that employed a total 140 participants who were selected using purposive sampling from the patients within the age group of 18–60 years old at Tehran in 2016 found that *Helicobacter pylori* infection increases the prevalence of memory and executive dysfunction (Rezvani et al., 2017). To the best of our knowledge, our study is the first to demonstrate the corresponding associations of selected taxa with specific domains of cognitive functioning among hypertension patients. Although a possible causal relationship between these selected taxa and different aspects of cognitive functioning deserves further investigation, our results promote the possibility that the precision intervention may be achieved by targeting specific bacteria associated with distinct cognitive dysfunction of hypertension patients.

To date, the current diagnosis of neurocognitive disorders mainly relies on neuropsychological scales and expensive neuroimaging. Exploration of potential objective and cost-effective indicators of neurocognitive decline is still limited. In the current study, we aimed to establish an objective and no-invasion prediction model based on selected gut bacteria to predict mild cognitive impairment of hypertension patients. To choose the optimal model for diagnosis of cognitive impairment of hypertension patients, the area under the ROC was analyzed. Our results showed that the bacteria-based multi-index prediction model (9×) performed best on mild cognitive impairment in hypertension patients (AUC = 0.944). Additionally, the single index prediction model based on *Escherichia–Shigella* has also promised efficiency prediction (AUC = 0.783), despite the accuracy being less than that of the multi-index prediction model. However, because a single intestinal flora is more efficient, this single index prediction model might also be valuable in applications.

Nevertheless, our studies have some limitations. Firstly, we only recruited a limited number of participants, making it possible to ignore the slight alteration of the intestinal microbiota. Secondly, it is needed to recruit a new cohort to verify the accuracy of our prediction models. In future experiments, we will expand the sample size and conduct a longer duration of follow-up visits for further verification of the results. Thirdly, 16S amplicon sequencing is not enough for functional analysis, and microbiome metagenomics should be carried out to improve the resolution of the taxonomic functional composition of the microbiome.

## Conclusion

Evidence demonstrated that hypertension-associated cognitive impairment and AD have overlapped pathophysiology. Effective diagnosis and early intervention for this neurocognitive decline could prevent the incidence of AD. The present study provides evidence for the involvement of intestinal microbiota dysbiosis in the cognitive impairment of hypertension patients. Moreover, we established efficiency prediction models based on selected taxa that strongly correlated with cognitive function performance. This work exposed a sensitive and objective index for the mild cognitive impairment of hypertension patients, and elucidate targets for preventing or reversing intestinal microbiota dysbiosis for future no-pharmacology therapeutics.

## Data Availability Statement

The datasets presented in this study can be found in online repositories. The names of the repository/repositories and accession number(s) can be found below: https://www.ncbi.nlm.nih.gov/, SAMN21436827-21436923.

## Ethics Statement

The studies involving human participants were reviewed and approved by Institution Review Board of Binzhou Medical University (No. 2018-085). The patients/participants provided their written informed consent to participate in this study.

## Author Contributions

YW and MC conceived and supervised the project. YW, MC, LQ, and YL designed and performed the main experiments. ZD, SM, LQ, and YL participated in 16S rRNA amplicon sequencing analysis. YL, LQ, WZ, ZW, CW, RM, XJ, and TZ recruited the subjects and collected samples. YL, ZD, and LQ drafted the manuscript. MC and ZD revised the manuscript. All authors have approved the final version of the manuscript.

## Conflict of Interest

The authors declare that the research was conducted in the absence of any commercial or financial relationships that could be construed as a potential conflict of interest.

## Publisher’s Note

All claims expressed in this article are solely those of the authors and do not necessarily represent those of their affiliated organizations, or those of the publisher, the editors and the reviewers. Any product that may be evaluated in this article, or claim that may be made by its manufacturer, is not guaranteed or endorsed by the publisher.

## Footnotes

1
https://www.genome.jp/kegg/genome/

2
https://www.arb-silva.de/documentation/release-138/
